# Mapping the role of structural and interpersonal violence in the lives of women: implications for public health interventions and policy

**DOI:** 10.1186/s12905-015-0256-4

**Published:** 2015-11-11

**Authors:** Stephanie Rose Montesanti, Wilfreda E. Thurston

**Affiliations:** 1 School of Public Health, University of Alberta, 11405-87 Ave, Edmonton, AB T6G 1C9 Canada; 2 Department of Community Health Sciences, Faculty of Veterinary Medicine, University of Calgary, Calgary, Canada

**Keywords:** Structural violence, Interpersonal violence, Women’s health, Scoping review

## Abstract

**Background:**

Research on interpersonal violence towards women has commonly focused on individual or proximate-level determinants associated with violent acts ignores the roles of larger structural systems that shape interpersonal violence. Though this research has contributed to an understanding of the prevalence and consequences of violence towards women, it ignores how patterns of violence are connected to social systems and social institutions.

**Methods:**

In this paper, we discuss the findings from a scoping review that examined: 1) how structural and symbolic violence contributes to interpersonal violence against women; and 2) the relationships between the social determinants of health and interpersonal violence against women. We used concept mapping to identify what was reported on the relationships among individual-level characteristics and population-level influence on gender-based violence against women and the consequences for women’s health. Institutional ethics review was not required for this scoping review since there was no involvement or contact with human subjects.

**Results:**

The different forms of violence—symbolic, structural and interpersonal—are not mutually exclusive, rather they relate to one another as they manifest in the lives of women. Structural violence is marked by deeply unequal access to the determinants of health (e.g., housing, good quality health care, and unemployment), which then create conditions where interpersonal violence can happen and which shape gendered forms of violence for women in vulnerable social positions. Our web of causation illustrates how structural factors can have negative impacts on the social determinants of health and increases the risk for interpersonal violence among women.

**Conclusion:**

Public health policy responses to violence against women should move beyond individual-level approaches to violence, to consider how structural and interpersonal level violence and power relations shape the ‘lived experiences’ of violence for women.

**Electronic supplementary material:**

The online version of this article (doi:10.1186/s12905-015-0256-4) contains supplementary material, which is available to authorized users.

## Background

Theoretical explanations for violence against women have developed in an attempt to understand the factors influencing violent acts. Studies that focus primarily on individual and proximate determinants of violence, such as domestic and other gender-based violence [1, 2], excludes the broader contexts and inequalities that lie at the root of multiple forms of violence in the lives of women [2–4]. Such violence, often referred to as interpersonal violence refers to everyday violence on a micro-interactional level such as sexual or physical abuse or assault that can occur either between family members, intimates, acquaintances or strangers [5]. While not discounting the significance of this research, an emphasis on individual or proximate-level determinants associated with violent acts ignores the roles of larger structural systems that shape interpersonal violence, thereby omitting economic, legal, and political factors; all of which are known to have important roles in determining a woman’s health [2]. Structural forms of violence are invisible manifestations of violence or any harm that are built into the fabric of society—political and economic organization of our social world—and creates and maintains inequalities within and between different social groups, gender and ethnic-cultural groups [6, 7].

**Fig. 1:**
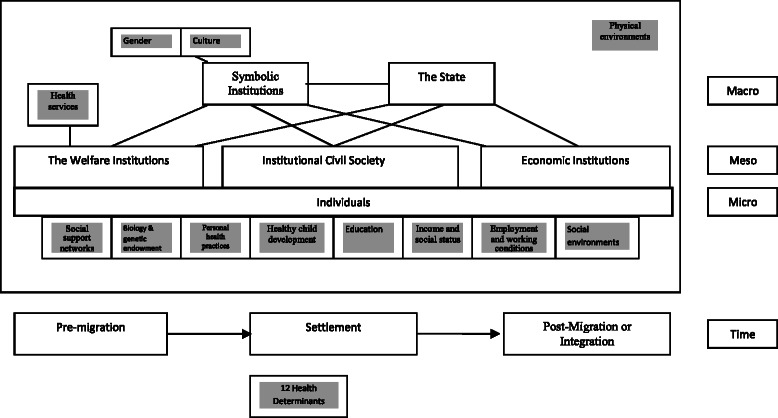
Thurston and Visandjée [12] Ecological Framework to Study Women’s Health. The framework illustrates the interplay of personal, situational, and sociocultural factors that shape women’s health. It offers a holistic approach to analyze multilevel and interactive influences of violence against women. In Canada, 12 determinants of health have been adopted in official policy: social support networks; biology and genetic endowment; personal health practices and coping skills; healthy child development; education; income and social status; employment and working conditions; social environments; physical environments; health services; gender; and culture [23]. These determinants are shaded in boxes. Gender and culture are listed in Symbolic Institutions. The micro-level is that of the individual woman who embodies the meso- and the macro-systems or institutions. Gender is shaped by micro-level politics: gender expectations, gender norms, a socially constructed body, symbolic representation and symbolic language. Gender also orders and is ordered by other social institutions at the meso- and macro-levels: the economy; ideologies; family; politics; religion; and the media [20]

Feminist theorists have focused on male-dominated social structures and socialization practices that teach men and women gender-specific roles that can influence violence and abuse against women [8]. In the past decade or so, scholars have argued that a complete understanding of violence against women requires acknowledging factors operating on multiple levels [9]. Ecological frameworks, core to population health promotion [9–12], have been applied to studies on violence against women to demonstrate that there are factors exogenous to individual women that interact to increase their vulnerability to violence. An ecological model adapted by Thurston and Vissandjée [12] is particularly useful for understanding the interplay of personal, situational, and sociocultural factors that shape violence against women and impact their health. This model calls attention to the known determinants of health within the context of structural variables, especially the operation of gender and other social institutions, and the social and physical environments, which can influence and perpetuate interpersonal violence [12] (Fig. 1).

In order to re-conceptualize a public health response beyond individual-level approaches to violence, we need to consider how structural and interpersonal level violence and power relations interact to shape the ‘lived experiences’ of violence for women in varying situational and cultural contexts [13]. In this paper we discuss the findings from a scoping review that examined how structural or systemic violence contributes to interpersonal violence against women. We used concept mapping to illustrate what was reported on the relationships among individual-level characteristics and structural level influences on violence against women and the consequences for women’s health. Our analysis draws on the work of Geoffrey Rose’s work (2001) on the “causes of the causes” of population illness to understand the relationship of the causes of violence against women [14]. We also follow the work of feminist theorists who have focused on male-dominated social structures and socialization practices that teach men and women gender-specific roles that can influence violence and abuse against women [8].

### Conceptualizing gender, structural, and symbolic violence

#### Gender as a symbolic institution

Gender is understood as a constitutive element of social relations based upon perceived (socially constructed and culturally variable) differences between females and males, and as a primary way of signifying (and naturalizing) relationships of power and hierarchy [15]. All social interactions and the social institutions in which these relationships occur are gendered is some manner. To say that a social institution is gendered means that constructions of masculinity and femininity are intertwined in the daily life of political, economic and legal institutions [16]. Gender relations are reproduced and reinforced through daily social interaction [16]. Gender as a social institution organizes social life in hierarchical, mutually exclusive categories, which maintains subordinate positions, whether material or ideological, among people within families, households or communities [17]. The term “gender-based” is used in international policy statements to highlight that violence against women is shaped by gendered arrangements of power in society. The UN Declaration was the first international statement that defined violence against women within a gender-based framework and identified the family, the community and the state as major sites of gender-based violence (GBV) [18]. GBV is a deliberately broad term in order to recognize the gendered elements in nearly all forms of violence against women and girls, whether it is perpetrated through sexual violence or through other means. “Gender-based violence” is also used in a more inclusive sense of referring to violence that is in some direct way concerned with expressing and maintaining the unequal power relations of oppressive gender orders. This includes not only violence against women, but also violence against men, transgender or transsexual individuals.

#### Structural violence

Structural violence refers to the social arrangements that put people and populations in harm’s way. The concept of structural violence has been used to explain multiple vulnerabilities globally [6, 7]. Structural violence is built into the fabric of society—political and economic organization of our social world—and creates and maintains inequalities within and between different social groups, and also among ethnic-cultural or other minority groups (referred to as ethnicity and minority-based structural violence). In contrast to physical violence, structural violence is invisible and can manifest itself indirectly [2]. Rather than focusing on dichotomized notions of ‘victims’ and ‘perpetrators’, which locate the problem of violence within individuals who are deemed good or bad, violent or non-violent, our attention to structural violence directs us to examine the “everydayness” of violence from the vantage point of complex political, social, historic, and economic processes. Structural violence is expressed in unemployment, unequal access to goods and services, and exploitation, which impacts a range of determinants of health. Lenon [4], for instance, highlights the devastating effects of the cutbacks to social services for abused women in Ontario. Structural violence was manifested in the form of crumbling social support programs and polices that had provided essential support to women in violent situations, and which forced some women to return to their abusive situation (p.g. 403).

#### Symbolic violence

Similar to structural violence, symbolic violence is an invisible mode of domination. Symbolic violence refers to the ideologies, words, nonverbal behaviors or communications that express stereotypes, hegemonies and create humiliation or stigma. Symbolic violence draws from other social institutions (e.g., the family, religion, education, economic and political intuitions) and is therefore often constructed and named as normal and natural. It, therefore, reproduces and perpetuates patterns of inequality and marginalization of women. For instance, symbolic violence is manifested in how gender roles are discussed, portrayed or rewarded and reinforces gender expectations, legitimizing acts of domination towards women. Women internalize their gender roles, referring to what Bourdieu calls “the habitus.” The distinctiveness of symbolic violence lies exactly in the fact that ideologies, dominant discourse, language and words that subordinate and marginalize women are seen as part of “the social order of things” [19].

## An ecological framework to study violence against women

We used the ecological framework on health developed by Thurston and Vissandjée [12] (Fig. 1) as our conceptual framework for understanding the interaction between structural and interpersonal violence and the health of women. This framework incorporates macro (structural & symbolic institutions), meso (group), and micro (individual) levels of analysis, the idea of time and life course analysis, and the determinants of population health. In an ecological framework, violence against women is said to result from the interaction of systems at different levels. The micro-system includes relationships in the immediate context in which violence takes place, for instance, interpersonal violence that occurs within family and intimate or close relationships. The meso-system represents the interplay between various aspects of a person’s organized social environment. The meso-system includes linkages between an individual’s family and other ambits of involvement such as place of work, extended family, network of peers, or services in the community. Lastly, the macro-system encompasses the social institutions and social structures, both formal and informal, in which the other systems are embedded—e.g., the world of gender, social expectations, cultural practices, and identity groups.

At the micro-level the individual woman embodies the meso- and the macro-systems or institutions. Gender helps establish and is established by micro-level politics: patterns of expectations; processes of everyday life; a socially constructed body; self and identity; desire; symbolic representation; interactions among friends, kin and strangers; and language and symbolism [20]. Gender also orders and is ordered by other social institutions at the meso- and macro-levels: the economy; ideologies; family; politics; religion; and the media [20]. Hence there is not one gender, rather gender is a performance that people enact in given contexts according to their perceptions, needs and desires.

## Review question

The research questions guiding the scoping review were: 1) what role do social systems and social institutions (e.g., family, culture, education, economy, polity) play in perpetuating interpersonal violence against women?; and 2) how does structural and interpersonal violence intersect to shape the determinants of women’s health?

## Methods

We used a scoping review method to conceptually map and identify gaps in the literature related to structural and interpersonal violence against women. We sought primarily to map the key concepts underpinning structural and interpersonal factors that contribute to violence against women. Scoping reviews are conducted to examine previous research activity, disseminate findings, identify gaps in the research and/or determine the value of conducting a full systematic review [21, 22]. We conducted the scoping review using an iterative process that allowed for flexibility in the search, reviewing and conceptual mapping concepts as recommended by Arksey & O’Malley [21] and Levac et al. [22]. The research presented in this paper is a review of the scholarly literature. There was no involvement or contact with human subjects, and institutional ethics review was therefore not required.

### Systematic search strategy

We developed a list of search terms in consultation with a research librarian. Different combinations of terms were used to carry out several searches within each database. We searched the following electronic databases: Ovid Medicine, Nursing, Social Work, Women’s Studies, Social Sciences, and Psychology. We then identified a total of 10 relevant electronic databases relevant to these disciplines: Ovid Medline, PsychINFO, Social Work Abstracts, Social Services Abstracts, Family and Society Studies Worldwide, Family Studies Abstracts CINHAL, SocINDEX, Sociological Abstracts, and Psychology and Behavioural Studies. All searches were limited to English language, peer-reviewed papers published in a five-year period between 2007 and 2012. We limited the number of years to make the project feasible and to focus on most recent thinking. Search strategies were used as appropriate to the specific features of each database (Additional file 1). We identified 3169 abstracts that were imported into RefWorks^TM^, a reference management software, and 1176 duplicates of titles where identified in the software, leaving 1993 abstracts to review for inclusion and exclusion in our analysis.

### Paper selection

Papers were included if they had a discussion of: 1) social institutions (e.g., family, education, economy and polity); 2) social norms and practices (e.g., culture, gender, ethnicity, religion); and 3) the SDOH and its relationship to gendered violence. All countries and populations were included and review articles were not excluded as we did not require primary research.

Papers were excluded if: 1) it was an editorial; 2) authors addressed only prevalence without moving beyond individual level analysis (e.g., alcohol as an individual’s problem); 3) interpersonal violence outcomes were discussed but not in terms of social context around the outcomes (e.g., women with interpersonal violence are identified as more likely to have HIV but the why is not discussed); 4) research where the victims were men; 5) research where the victims were less than 18 years of age and there was no link to adult experiences; 6) research on help seeking behaviour of women when only individual characteristics are explanatory; and 7) book chapters. This process left 174 papers relevant for coding (Additional file 1).

### Data extraction and conceptual map analysis

We began with a full-text review of the empirical and non-empirical papers. We recorded descriptive information from the papers (where available) on the authors, year of publication, goal of the paper, study design, target population, sampling technique, and geographical location. The flow chart in Additional file 1 represents the different methods used in the studies included in the review. We also took note of any differences or contradictions in how structural and interpersonal violence were defined or applied in the paper.

### Coding process

We developed a template for coding the papers (Additional file 1) using Thurston and Vissandjée ecological framework [12] (Fig. 1). We added kinds of violence at the individual level; kinds of structural violence; immigration issues; Aboriginal issues; and causal theories explaining the links between structural and individual violence that were mentioned.

We selected the first five papers in RefWorks^TM^ and coded them separately using the template to assess whether there was good agreement between coders and if the template failed to capture sections of the papers. We used the Public Health Agency of Canada’s (PHAC) definitions of each determinant of health included in the framework [23]. We also added two outcomes (health and quality of life) that were not evident in the framework but were important to learning about health consequences. A student research assistant coded 174 papers and then the lead author and the research assistant created concept maps based on the coding for each of domains in the framework. We kept Health Services as part of Welfare Institutions provided by the State. Physical Environments were not mentioned enough in the literature to warrant a separate concept map.

As we proceeded we did not create a concept map for *Biology and Genetic Endowment* (identified as a determinants of health by PHAC) as there was little if any mention of such factors, apart from race, which was captured under ethnicity and minority status. We decided to keep *Culture* separate, recognizing that culture is imbued in everything in society, but discussed as a separate factor in the papers. We decided to do separate maps for *Income and for Social Status* as the papers indicated that social status was more than income and we included *Economic Institutions* with the *Income* map as that was mainly how they were discussed in these papers. Thus, we concluded with 11 domains in which the issue of structural violence was discussed. These concept maps indicated which and how many articles raised each concept and whether concepts might be related (e.g., education and ethnicity or culture). The authors discussed each concept map and identified common themes across the maps. As we proceeded with discussion and moved back and forth between the domains, between 2 and 4 drafts of concept maps were created for each of the 11 domains. We then reviewed each map for consistencies and created final drafts.

We then used Cmaps^TM^ software to create a single concept map that illustrates a “web of causation” of gender-based violence against women. A concept map is a diagram that depicts suggested relationships between multiple concepts. In our concept map, the concepts that were coded in our analysis are enclosed in the circles and are connected with arrows and linking phrases such as *contributes to* or *relates to* articulate the relationship between concepts. Mapping allows you to see gaps in knowledge and areas of contradiction.

## Results

**Fig. 2:**
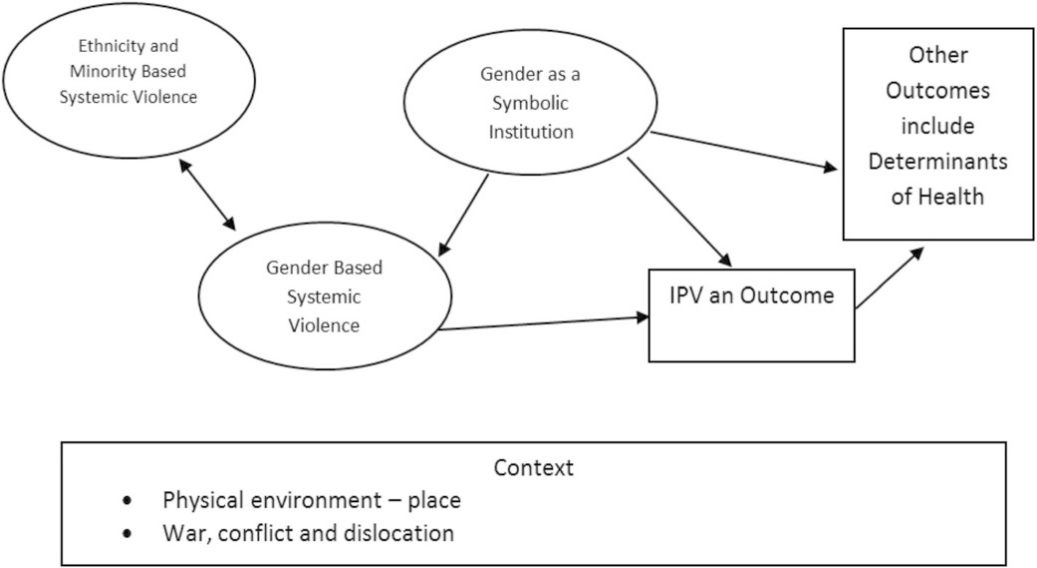
The Relationship between Gender-Based Structural Violence to Interpersonal Violence Experienced by Women. The framework illustrates how structural violence can lead to interpersonal violence against women. Gender as a symbolic institution (GSI)—e.g., gender role performance—may have a causal role in creating interpersonal violence

**Fig. 3:**
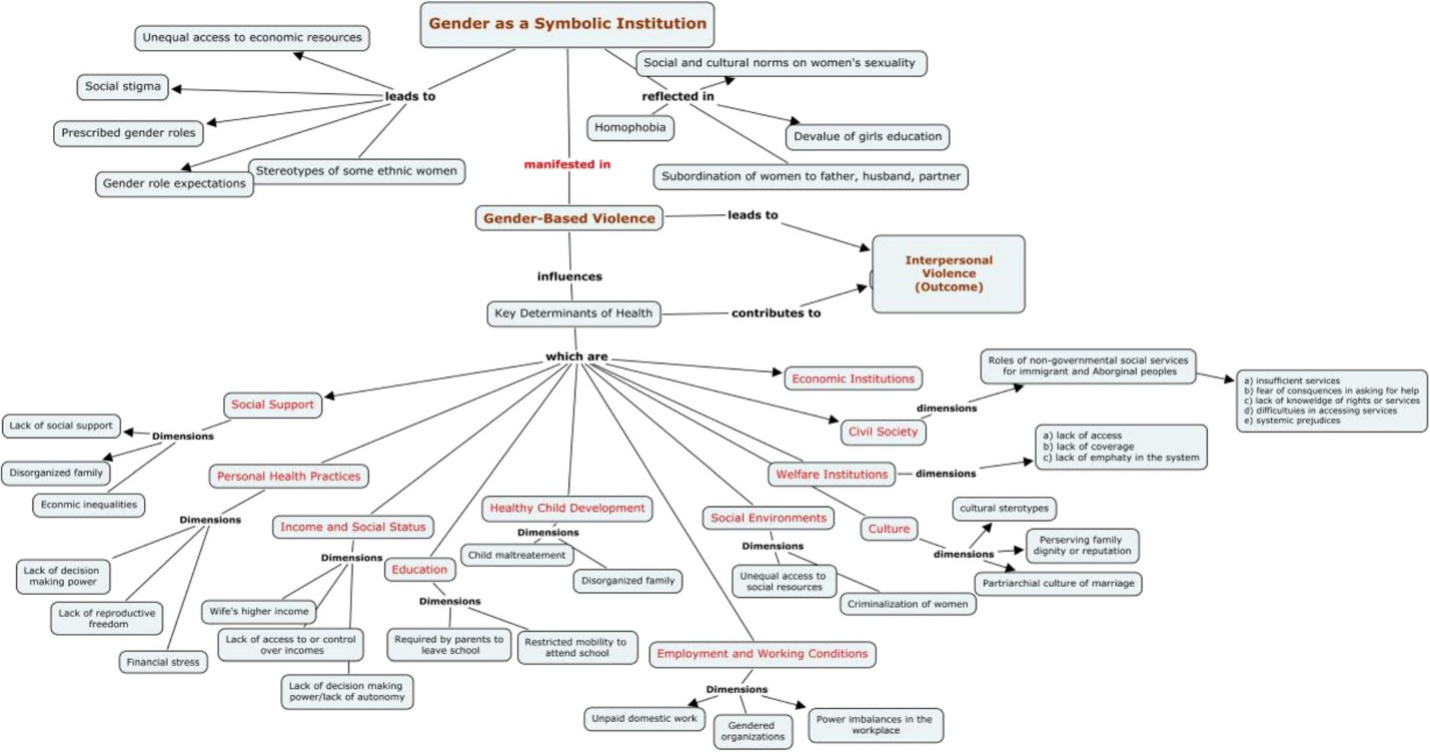
Concept Map: Relationship Among Individual-level characteristics and Population-level Influences on Gender-based Violence Against Women and Consequences on Women’s Health. Using the Cmaps^TM^ software we created a web of causation for gender-based violence against women that illustrates the interrelationship between structural and interpersonal violence for women

The literature was clear that structural or systemic violence can lead to interpersonal violence against women (Fig. 2). Gender as a symbolic institution (GSI)—e.g., gender role performance—may have a causal role in creating interpersonal violence. Our findings from the scoping review also demonstrate how structural factors affect the SDOH for women and can lead to violence against them. The research evidence shows that there is an interrelationship between the determinants of health and interpersonal violence. When the basic determinants of women’s health are not met, women can be susceptible to interpersonal violence, and likewise, the occurrence of interpersonal violence among women can further impact on those determinants. The consequence of violence on the determinants of health varies for women in different ethnic and cultural groups. Our concept map (Fig. 3) illustrates the interrelationship between structural and interpersonal violence for women. In the sections below we describe what is known about the possible links between the social determinants of health and interpersonal violence.

### Social support

PHAC identifies social support from families, friends and communities to be associated with better health outcomes. According to PHAC “social support networks are important in helping people solve problems and deal with adversity, as well as in maintaining a sense of mastery and control over life circumstances” [23]. Individual social support can include support from family, social networks, and from community groups and organizations whose members may provide support to a woman that is protective against further abuse or that mitigates the effects of abuse. However, a lack of social support can leave a woman in an abusive relationship [24].

Gender as a symbolic institution prescribes what is expected of a woman in her roles (i.e., submissive behavior, marriage obligation, male superiority), which shapes the kind and extent of individual and social support that women receive if they do not act in the roles expected of them [24–26]. On the one hand, close family ties in the family of origin, extended family and family of in-laws can result in positive social support for a woman [26–31]. On the other hand, such support is constrained by concomitant values of family privacy, unit, and maintenance of the family’s reputation [24–26], which means that individual family members frequently subordinate their needs to ensure family solidarity, which for some women might mean tolerating abuse [25, 26]. Thus, a woman who experiences interpersonal violence has a risk of losing further social support networks.

Structural violence limits the opportunity structure of individuals through the unequal distribution of resources and lack of economic opportunities, which can result in disorganized families [32, 33]. Structural violence reproduces social relations of exclusion, marginalization and oppression (e.g., intergenerational trauma) and has negative consequences for physical and emotional well-being (e.g., poor family relations, substance abuse) [32, 34]. Such disruptions within the family, therefore, can immediately put a woman at risk of having less social support if these consequences are not remediated [32]. Immigrant women are also more likely to be isolated from their family networks and experience difficulties in access to services in the host country because of lack of knowledge, immigration status, and language barriers [35, 36]. Ethnicity and minority-based structural violence results from strong taboos, stigma, and shame around sexuality in some cultures that constrain their decision-making power in relationships. For example, structural violence is manifested in forced marriage which influences a woman’s decision-making power in choosing her own partner and can put her at risk of abuse [37].

### Personal health practices and coping

According to PHAC, “personal health practices and coping skills refer to those actions by which individuals can prevent diseases and promote self-care, cope with challenges and develop self-reliance, solve problems and make choices that enhance health” [23].

The literature on interpersonal violence examines personal health practices and coping in relation to help-seeking behavior among abused women. Symbolic violence in the form of a woman’s subordination to men, either their father or husband, can result in controlled access to health and social services and limits a women’s decision-making power and autonomy. Ideologies about gender roles and expectations are so pervasive within the family, work, and the welfare system, that women internalize their gendered subordination to men as natural and blame themselves for their conditions hindering them from personal health practices.

Furthermore, research also demonstrates that women who earn higher incomes or who are financially independent from their partners are more likely to seek help from abusive or violent relationships [38]. There were limited discussions in the literature on how other socio-demographic factors (e.g., culture, ethnicity) influence a woman’s help-seeking practices.

### Education

PHAC identifies health status to improve with level of education. According to PHAC, “education is closely tied to socioeconomic status, and effective education for children and lifelong learning for adults are key contributors to health and prosperity for individuals, and for the country” [23].

The literature presented an education paradox for women, where educational attainment can be both a protective factor and contributor to interpersonal violence. The papers we reviewed that refer to the education of girls and women in particular contexts noted that equal education for both sexes was shown to be threatening to men’s power both in personal relationships and society. For instance, in one study, lower education of women was associated with fewer incidents of violence [39]. This is congruent with the notion that gender-based violence reproduces and maintains the status quo in terms of gender equality. In other studies, however, having a higher education was viewed as a protective factor for women. Women with a higher education were described to be more likely to think strategically in accessing resources and escaping violent relationships [40]. One study highlighted the need to examine gender inequities in terms of the lifecycle of girls and women. For instance, young women in abusive relationships that resulted in pregnancy usually dropped out of school, and therefore, had fewer economic opportunities for employment [41].

The way symbolic violence manifests itself through gender is evident in practices where parents require girls to leave school to help at home [39], or in families that devalue the education of girls. Systemic and symbolic violence is also present in ethnic and cultural structures. The papers that examined educational attainment and the risk of gender-based violence did so within one racial or ethnic population [41–44]. There was limited discussion in the papers on policies and interventions to support women’s education.

### Healthy child development

Early child development is identified by PHAC as an important determinant of health in that other determinants of health (e.g., family income, parents’ education, housing quality, access to nutritious foods, etc.) affect the physical, social, and mental development of children and youth [23]. Children’s early physical and psychological development depends mainly on the social environment and how it fosters the child’s social interaction with others. Children who are victimized or witness some form of abuse may have emotional, behavioral, cognitive, social and psychological developmental deficits [45].

Gender-based structural violence is represented by maltreatment of young girls [43, 46–48] in the form of physical, emotional, and sexual abuse. Children who live in disorganized families (e.g., abandonment by a parent, parental alcohol and/or drug abuse, foster care) are more likely to witness adult violence [26, 47, 49] and experience violence (e.g., rape, sexual assault or abuse) throughout their lives. As a result, children in a disorganized family may in turn form a disorganized family of their own. Research also demonstrates that the outcomes of childhood abuse can be detrimental to their life chances and opportunities later in life. For instance, victims of childhood abuse used marriage as an escape from their abusive families, which only put them at an increased risk of interpersonal violence in adulthood [41] and they sometimes became dependent on alcohol and drugs as a coping strategy.

Research has also demonstrated that some cultural norms and practices have emotional, physical and mental health consequences for girls in their adulthood [50]. Structural and symbolic violence is represented through cultural norms and practices that discriminate against girls, such as female genital mutilation, forced marriage, dowry, and honor killing, and pose a violation of human rights in some countries. Structural violence, through the unequal distribution of resources and lack of economic opportunities, is also demonstrated by the forced entry of young children from economically marginalized or disorganized families into sex work, which puts them at risk of sexually transmitted infection and mental health problems [51]. Homelessness may be another outcome for children and youth from disorganized families [2].

### Social status

According to PHAC, “there is strong and growing evidence that higher social and economic status is associated with better health” [23]. We described that gender as a symbolic institution is reproduced by power imbalances in the family and intimate relationships. Prescribed gender roles discourage women from paid employment and contribute to economic dependence of women on men [27, 28, 42, 52, 53]. Marital conflict may result from expectations about gender roles, household chores and domestic duties, and may trigger feelings of diminished control by the husband [27, 28, 42, 52–54]. Lack of access to or control over incomes occurs when a woman has no financial independence when she brings in family income. Unequal participation of women in labour markets is also influenced by women’s access to a work place (e.g., rural setting, transportation barriers), education, and availability of resources [55, 56].

Ethnic and minority-based structural violence can put women in some ethnic groups or cultures at a greater disadvantage in finding employment [29, 41, 44, 57, 58]. Among the factors described in the literature that produce differential opportunities for women of some cultures or ethnicity include: 1) lack of recognition of foreign credentials; 2) undocumented immigration status; 3) laws that specify dependency and preclude separation or divorce; and 4) ability to integrate (e.g., get a drivers’ license); and 4) differential treatment by Justice and Service Systems (e.g., negative stereotypes of First Nation women as sexually available, systemic prejudices by Government, police services, or justice systems) that place minority women as very low in social status in society [53, 59].

### Employment and working conditions

According to PHAC “unemployment, underemployment, stressful or unsafe work are associated with poorer health. People who have more control over their work circumstances and fewer stress related demands of the job are healthier and often live longer than those in more stressful or riskier work and activities” [23]. Workplaces are gendered institutions in that there are different expectations of men and women and an imbalance of power that favours promotions among men in the job. Women with children and domestic roles have a more difficult time balancing work responsibilities [47, 60, 61]. Lower wages for women is a form of gendered discrimination [30]. The dominance of male owners and employers is another indicator of gender-based systemic violence.

Furthermore, ethnicity and minority-based structural violence is represented in racial and cultural discrimination among women of colour and Aboriginal women by employees in the work place [62, 63]. Colonization of Aboriginal peoples, which included a history of residential schools that aimed at training those generations to do agricultural and service work, has left many in low-paying jobs or living in poverty [52].

### Social environment

PHAC identifies a supportive society to reduce potential risks to individual health. Factors related to the social environment include civic vitality of the strength of social networks within a community, region, province or country; social stability and cohesive communities. Research has demonstrated the role of structural violence in undermining the social environment of women. Among the papers included in our review cultural norms and institutions maintain social constructions of gender. Social and cultural norms of male violence and cultural norms of patriarchy and male dominance, social stigma and shame of divorce, exercise of control by in-laws, and economic dependency on husband/spouse put women in more vulnerable positions.

The scholarly literature highlights how cultural norms and institutions maintain gender. Social and cultural norms of male dominance and acceptance of violence, social stigma of divorce, and economic dependency on husband/spouse put women in a more vulnerable position to violence [64]. Gender-based violence is represented in women’s lack of decision-making power, lack of reproductive freedom, and obstacles in access to social resources or access to economic resources, or access to services, such as health care [27, 47, 64].

Research shows that ethnic-minority women are at an increased vulnerability to discrimination in employment, education, access to housing, and other social and economic resources, and face increased barriers to financial independence [65]. Poverty and economic insecurities push women from some cultures to early marriage, negative relationships, polygamous marriage, or sex work [46, 64], therefore, reproducing ethnicity and minority based structural violence. For instance, many women, for instance, are forced to stay with the abusive husbands because of the bride price paid to the parents or threat of abduction of children if the women leave the marriage [46]. Immigrant women face subordination not only as a woman, but also as a minority woman in a foreign land. Lesbian women are also minorities, and experience a loss of social networks and ties in their community, experience physical assault by family members, and are denied equal opportunities by co-workers because of their sexual orientation [66].

### Culture

According to PHAC’s description of culture, the underlying premise is that: some persons or groups may face additional health risks due to an environment which is largely determined by a dominant set of cultural values that contribute to the perpetuation of conditions such as marginalization, stigmatization, loss or devaluation of language and culture practices and lack of access to culturally appropriate health care and services [23]. In our analytic framework, we see this as better described as a process of racialization and stereotyping by the most powerful, who may not even be the largest groups [9, 16].

Gender-based structural violence was discussed in terms of patriarchal rules of marriage [27, 28] and women’s sexuality in terms of female genital mutilation, and lack of decision making power in sexual relations. A 2003–2007 meta-analysis of interpersonal violence using Demographic and Health Survey data in 17 Sub-Saharan African countries found that women were more likely to justify wife beating than men and that sex disparities in attitudes towards interpersonal violence increased with the practice of polygamy. The meta-analysis also found that the magnitude of sex disparities in interpersonal violence attitudes decreased with increasing adult male and female literacy rates, gender development index, gross domestic product, and human development index [67].

### Civil society

Non-profit organizations “provide services and opportunities…and act through individuals and collectivities, and between collectivities and the state” [12]. Gender-based violence was discussed in terms of the roles of non-governmental social services and services for immigrant and Aboriginal peoples and how they might perpetuate rather then address the problem of violence against women. The papers on social services were predominately about shelters for women fleeing domestic violence. These papers noted insufficient services [24, 27, 57, 68]; lack of trust in workers and fear of consequences of asking for help [28]; difficulties in accessing services (i.e., transport cost and inability to contact service providers while they lived with the abusive families) [28, 69]; lack of knowledge of rights or services [35, 54] and systemic prejudices by government and society [33]. On the positive side there was recognition that shelters for women play an important role [29] in offering physical protection in safe housing, legal constraints against the perpetrator, and training based on the rights of women.

The papers that touched on ethnicity and minority-based structural violence noted that minority women lacked knowledge of laws existing to support them from abuse, availability of resources, and their entitlements to services [24]. The literature identified the need for culturally appropriate services [25, 60], limited availability of translators [31] and lack of services in their first language [24]. Some papers reported that immigrant women experienced reasons to distrust welfare institutions [24], culturalization (e.g., assumed *cultural* acceptance of wife abuse) [46, 60, 70], and racism and discrimination [53].

## Discussion

The different forms of violence—symbolic, structural and interpersonal—are not mutually exclusive, rather they relate to one another as they manifest in the lives of women. Structural violence is marked by deeply unequal access to the determinants of health (e.g., housing, good quality health care, and unemployment), which then create conditions where interpersonal violence can happen and which shape gendered forms of violence for women in vulnerable social positions. Our web of causation illustrates how structural factors can have negative impacts on the social determinants of health and increases the risk for interpersonal violence among women. For instance, interpersonal violence is more likely to occur in relationships where a relatively large income gap exists between the partners [38]. Hence, not having their own income might not only place women at risk of financial dependence on their partners, but might also place them at risk of being repeatedly victimized by their intimate partners [38]. In contrast to income and employment status of women, the education of women was described to both influence violence or abuse and protect women from interpersonal violence from men. A higher level of education, therefore, might help an abused woman to identify and access appropriate services in the community once the abuse has started. Other things being equal, this could mean that an educated woman might be less likely to remain in an abusive relationship, but the mediation of other sources of structural and symbolic violence cannot be underestimated.

Gender is a critical determinant of health, affecting a person’s access to, and control over financial and physical resources, education and information and freedom of movement. Gender as a symbolic institution interacts with other social and economic institutions, creating and maintaining risk of violence for women.

Our findings resonate with the work of others who have demonstrated the relationship between the social determinants of health and interpersonal violence. Scholars have theorized that relative deprivation, a meso-level variable, causes micro-level stress through a decreased sense of self-worth and a lack of autonomy, which can in turn lead to violence [71] that is acted out in very gendered ways (men towards spouses, fathers towards children). However, extensive research is still needed to fully understand the relationship between the social determinants of health and victimization of women by intimate partners. It is clear that many of the relationships are complex and not simply linear nor unidirectional. In large-scale studies with a sample size sufficient to do path analysis, we may gain greater clarity on the relationships [72]. If we think about inadequate and unsafe housing for instance, we can postulate that substandard and unsafe housing conditions can be both a consequence of and a contributor to interpersonal violence. To plan population health promotion to prevent interpersonal violence, we need to be clear whether it is a cause or an outcome. Clearly, sexual violence or abuse from an intimate partner can be a contributor to inadequate housing arrangements or even homelessness among women who leave an abusive relationship [4]. We may also hypothesize following some stress theories that certain housing arrangements (like multiple families living in the same dwelling, as is the case among on reserve Aboriginal evacuees from the June 2013 floods in Southern Alberta), might generate stress that can ignite incidences of interpersonal violence; however, are these incident or prevalent cases in reality? Policies and interventions to prevent and address incidences of interpersonal violence, therefore, need to understand the “causes of the causes” that create and maintain gender inequities and gender domination, which put women at a greater risk of violence, abuse or harm from intimate men.

Further evidence from evaluation research is also needed on effectiveness of interventions and policies that address interpersonal violence among women. While attempts have been made to pay closer attention to gender as a determinant of interpersonal violence (e.g., gender mainstreaming in policy), there is limited analysis aimed at understanding the systemic factors that cause and maintain gender inequality, putting women at higher risk of violence or abuse. Violent acts by male perpetrators need to be examined and understood in the context of structural inequities that cause and maintain gender inequality (e.g., rape myths). For researchers, engaging a structural violence analytical framework offers the possibility of overcoming conventional dichotomies of victims and perpetrators, or violent and non-violent actors [73], instead revealing how norms of subjugation are enacted, accepted, questioned, challenged, neglected, and also habitually perpetuated, influencing the behaviors of both parties in relationships. Gender analysis highlights important differences in the form and consequence of violence experienced by men and women with clear implications for health policy. Addressing the role of structural violence in women’s health inequities has the potential, therefore, to advance knowledge in primary, secondary and tertiary prevention of interpersonal violence from a population health perspective.

There is also insufficient examination of GBV against Aboriginal peoples in the scholarly literature. Further research is needed to understand interpersonal violence against Aboriginal women in the wider context of the family, community and social institutions that have constructed the problem and maintain the higher than average rates. Also, while research on violence against Aboriginal women is important, not elucidating the roles of masculine and Aboriginal identities is missing a key aspect of the causes of the causes. It is insufficient to continue to only assess risks for interpersonal violence at the individual level. Men’s health is then also neglected in discussions of interpersonal violence against women [52].

Violence against women has been identified as a serious public health issue globally; therefore, effective interventions to address GBV should be situated within the Ottawa Charter model of health promotion, which encompasses personal, social, and environmental well-being. Having focused on individual level causes, the field has neglected the key elements of the Ottawa Charter for Health Promotion that have not been inadequately applied to the prevention of violence among women. The Ottawa Charter [74] identified five key elements to promote peoples’ health that are important in the prevention of violence against women. These key elements include: building healthy public policy, creating supportive environments, strengthening community action, developing personal skills and reorienting health services. Creating supportive environments for abused and assaulted women may include public policies to promote living and working conditions that are safe, non-discriminating, and equitable towards women. These could include employee assistance programs that explicitly recognize that both victims and offenders may seek individual assistance for prevention of interpersonal violence [75]. Intervening at systemic levels to implement healthy public policies that promote the health, social and economic prosperity of women can empower women to have greater control of the factors that influence their livelihoods, foster their autonomy and decision-making power. In providing opportunities for women in employment and education, women develop the personal skills to understand the consequences of violence and how to promote and protect their own health.

Reorienting health and social services for abused or assaulted women involves the development of culturally competent and culturally safe services [76] that can respond to abused women’s needs across ethnicity and culture. Abused women, who come from different religious and cultural backgrounds, or sexual orientation, require the ability of service providers to work with women to untangle what are valued traditions of a culture and what are the social institutions that maintain inequities. Often, working with other women to gain what Friere [77] called conscientization can be helpful. At the very least, service providers need to understand that everyone is embedded in cultures, not just those who may be the ‘other.’ Cultural competence requires an understanding of our own social institutions as well as the particular cultural needs that different communities have [76, 78].

A strength of this study was the use of concept mapping to examine the relationships among the factors that contribute to interpersonal violence against women. This approach incorporates the complexity of a wicked social problem, one that has been under serious scrutiny for at least three decades in North America, but with little reduction in the prevalence. Whereas the structure of other qualitative approaches, such as focus group discussions, often results in consensus and discussion regarding a single theme, concept mapping allows for the exploration of multiple themes at the same time and for a better understanding of how those themes are related to one another.

## Conclusion

By drawing attention to structural and symbolic forms of violence we understand that violence does not come solely from interpersonal relations so it may be time to find a label other than *interpersonal* to describe what is happening. The recent attention in Canada to the number of Aboriginal women who have *disappeared* with little if any resulting investigation by justice, welfare, or other institutions [79, 80] illustrates this very well as does the relatively little attention in the published literature on the experiences of gay, lesbian, bisexual, transgendered and transsexual (GLBTT) populations. It is accepted that the individual, the agents who perpetrate and experience violence must not be forgotten [12], but the challenge moving forward is to work from a population health perspective to prevent structural violence against women. This is not a new concept, but has been a key aspect of feminist analysis of violence against women for the three decades [81]. This analysis must be renewed with a deeper understanding of masculinities and gender-based violence (GBV).

The accounts of the daily lived experiences of abused or assaulted women are still important as they can highlight the intersections of micro, meso and macro levels in producing and reproducing violence among women. Through this lens, we see how women experience interpersonal violence not only in direct, physical harm, but also though the injuries that come from the bureaucracies within institutions that do not respond to their needs and instead disrespect and mistreat them and further exacerbate their marginalization. Interpersonal violence against women is multidimensional problem with no single satisfactory explanation. Public health has been successful in reducing the prevalence of many complex health problems, but only when a population health lens has been used [13, 29]. This study suggests that public health strategies that fail to address structural violence and gendered power relations will continue to fall short in stemming the multiple harms that contribute to violent acts that occur among intimates.

## Additional file

Additional file 1: **Search Results.** (DOCX 201 kb)
