# Supplementary material for: Mapping the role of structural and interpersonal violence in the lives of women: implications for public health interventions and policy
Source: BMC Womens Health. 2015 Nov 11;15:100. doi: 10.1186/s12905-015-0256-4 (PMC4641364; doi:10.1186/s12905-015-0256-4)

Appendix A: Search Results

| **Medline**  **Limit** to English, Human, Female, 2007-2012,  577 results  **PsychINFO**  **Limit** to English, 2007-2012, Journal articles, Human  68 results  **Social Work Abstracts**  **Limit** to 2007-2011, English, all journals, women  73 results  **Social Services Abstract**  **Limit** to English, 2007-2012, Journal articles  343 results  **Family and Society Studies Worldwide**  Limit to academic journals, peer reviewed, English, 2007-2012, document type: Article,  337 results  **Family Studies Abstract**  Limit to academic journals, peer reviewed, English, 2007-2012  361 results; Limit to document type: Article  330 results  **CINHAL**  **Limit** to academic journals, published date 2007-2012, English, Peer reviewed, Sex: Female  391 results  **SocINDEX**  **Limit** to academic journals, peer reviewed, English, 2007-2012, document type: Article, Subject Thesaurus terms: Intimate partner violence, family violence, abuse of women, man-woman relationship, violence against women, abused women, marital violence, victims of family violence**.**  266 results  **Sociological Abstract**  **Limit** to English, 2007-2012, Journal articles  428 results  **Psychology and Behavioural Studies**  Limit to academic journal, peer reviewed, English, 2007-2012, document type: Article  325 results |
| --- |

Appendix: Coding Template

| Code |
| --- |
| Kind of Violence  Sexual, physical, mental, economic, etc. |
| Kind of Structural violence |
| Contribution of Structural to Ind Social Support |
| Contribution of Structural to Ind genetic & biology |
| Contribution of Structural to Ind PHP |
| Contribution of Structural to Ind HCD |
| Contribution of Structural to Ind Edu |
| Contribution of Structural to Ind Income |
| Contribution of Structural to Ind Social Status/Class |
| Contribution of Structural to Ind EWC |
| Contribution of Structural to Ind Social Enviro |
| Welfare Institutions – Health services/policies |
| Welfare Institutions – Social Service |
| Welfare Institutions –Legal services |
| Welfare Institutions – immigration |
| Welfare Institutions –First Nation/Aboriginal |
| Economic Institutions |
| Civil Society |
| Symbolic – Gender |
| Symbolic – Culture |
| Symbolic –Religion |
| Symbolic –Ethnicity/race |
| The state – laws |
| The state |
| Causal theories or links |
| Outcomes on Health |
| Outcomes on Q of L |

Appendix C: Methods used in review process


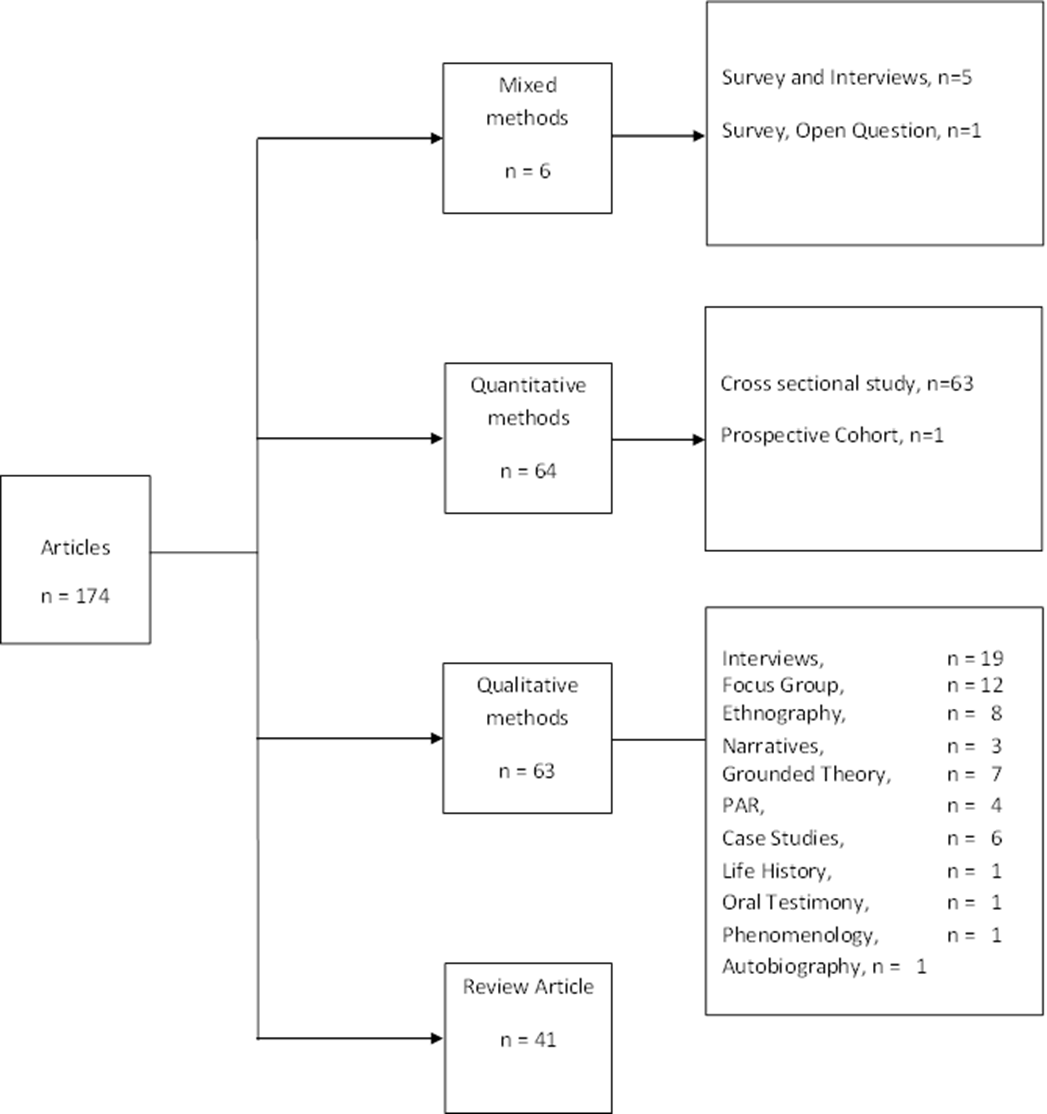

Supplement: Additional file 1: — Search Results. (DOCX 201 kb) [file 12905_2015_256_MOESM1_ESM.docx]
